# The Role of 5-Fluorouracil in Preventing Recurrence After Enucleation of Odontogenic Keratocyst: A Case Report

**DOI:** 10.7759/cureus.44777

**Published:** 2023-09-06

**Authors:** Chuimee Gogoi Barua, Iqbal Ali, Ashish Tripathi, Arindam Malakar, Pranjit K Singha

**Affiliations:** 1 Oral and Maxillofacial Surgery, Career Post Graduate Institute of Dental Sciences and Hospital, Lucknow, IND; 2 Oral and Maxillofacial Surgery, Prabhu Dayal Memorial (PDM) University, Bahadurgarh, IND; 3 Dentistry, Sardar Patel Post Graduate Institute of Dental and Medical Sciences, Lucknow, IND

**Keywords:** mcs, carnoy's, kcot, 5-fu, okc

## Abstract

The odontogenic keratocyst (OKC) is known for its high recurrence rate and disputed treatment modalities. In this report, we review the literature elucidating the efficacy of 5-fluorouracil (5-FU) topical application for recurrent OKC, and discuss the management of an OKC with 5-FU after enucleation and a 12-month follow-up.

A 38-year-old female patient with an aggressive OKC in the right mandible underwent surgical curettage followed by topical application of 5-FU. Regular follow-up examinations for 12 months (radiological evaluation at three, six, and 12-month intervals) showed no signs of recurrence, with complete resolution of the cystic lesion and gradual bone regeneration. New bone formation was identified in the radiographic follow-up.

This case demonstrates the potential efficacy of topical 5-FU as a promising treatment modality for OKC, warranting further research and validation. A novel and successful therapy for OKC is the topical application of 5-FU. After enucleation, topical application of 5-FU efficiently treats OKC, leading to normal bone healing and regeneration without any adverse local or systemic effects.

## Introduction

The odontogenic keratocyst (OKC) is a fascinating and clinically challenging entity due to its aggressive nature, high recurrence rate, and controversial treatment modalities. It was first described as a separate clinical entity by Phillipsen and Reichart in 1956 [1]. It was reclassified by the World Health Organization (WHO) as a benign keratocystic odontogenic tumor (KCOT) in 2005 due to its potential for recurrence and association with genetic syndromes such as Gorlin-Goltz syndrome [2]. The most controversial decision in the 2017 classification was to switch KCOT back to OKC because the evidence for its neoplastic hypothesis was deemed insufficient [3]. Odontogenic keratocysts typically arise from remnants of the dental lamina and commonly affect the mandible, particularly the posterior region [4]. They often remain asymptomatic until they reach a significant size, involving the mandibular ramus and body. Radiographically, OKCs present as unilocular or multilocular radiolucencies, which can be confused with other jaw lesions such as ameloblastomas or dentigerous cysts [5]. Histologically, OKC can be classified into two subtypes: parakeratotic and orthokeratotic. These subtypes are characterized by the type of keratin produced and the appearance of the epithelial lining. In the parakeratotic subtype, there is an increased production of keratin, and the cells in the lining do not exhibit keratolytic granules. Additionally, cells in the parakeratotic subtype tend to slough into the keratin layer. This classification is important as it helps to further understand the characteristics and behavior of OKC lesions.

To establish an accurate diagnosis, a comprehensive evaluation, including a clinical examination, radiographic analysis, and histopathological examination, is essential. The optimal treatment strategy for OKCs remains a subject of debate, with recurrence rates varying widely from less than 10% to over 60% [6]. The optimal treatment for OKC remains controversial, and there is no general consensus on the most appropriate approach. Several treatment modalities have been proposed, including marsupialization, decompression, enucleation alone, enucleation with adjunctive techniques such as chemical curettage using Carnoy's solution, liquid nitrogen cryodestruction, peripheral ostectomy, and even jaw resection. Enucleation with chemical curettage using Carnoy's solution was initially favored due to its lower recurrence rate; however, the use of Carnoy's solution has declined following the FDA's prohibition of chloroform, an ingredient in the original formulation, due to its carcinogenic potential. Modified Carnoy’s solution (MCS) without chloroform is often used in place of the original Carnoy’s solution, although it is less effective [7].

Recurrence theories for OKCs include incomplete removal of the cyst lining, growth from satellite cysts or residual odontogenic epithelial rests, or the development of unrelated OKCs in adjacent regions of the jaws [8]. The recurrence rate is higher in cases with an associated infection, fistula, or bony wall perforation. Multilocular OKCs tend to have a higher risk of recurrence compared to unilocular ones. It is also suggested that the biological behavior of the lesion and the expression of proliferative markers play a role in recurrence [9].

Recently, a novel approach using topical application of 5-fluorouracil (5-FU) has gained attention for OKC management. It is an antimetabolite that targets proliferating cells and is used to treat basal cell carcinoma (BCC) and other malignancies. It is a recognized treatment for actinic keratosis [10]. Based on molecular research on the protein patched homolog (PTCH) tumor suppressor gene pathway, a focused therapeutic strategy for BCCs has been developed. It is known that OKCs also arise from PTCH gene mutations similar to BCC [11]. Neoplastic growth is a result of PTCH1 mutations activating the smoothened (SMO) and sonic hedgehog (SHH) pathways. Rui et al. [12] reported that OKC development is likely impacted by SMO gene changes. This study raises the possibility that blocking SHH signaling pathways and suppressing SHH transcription factors could be an effective strategy to molecularly target OKC. In patients with nevoid basal cell carcinoma syndrome, the oral use of the SHH inhibitor vismodegib has proven to reduce the number and morbidity of numerous BCCs and OKCs [13].

The 5-FU stops the enzyme thymidylate synthetase (TS), which is necessary for the synthesis of DNA and results in cell death. Although the exact mechanism of action is unknown, 5-FU has been linked to a decrease in both the production of arachnoid acid metabolites and inhibition of apoptosis, as well as an increase in immune surveillance and anti-angiogenesis, thus impacting the cancer cells' capacity for invasion [14]. It acts in various ways, but its main role is acting as a TS inhibitor, stopping the enzyme's synthesis of the pyrimidine thymidine needed for DNA replication. Deoxyuridine monophosphate (dUMP) is methylated by TS to produce deoxythymidine monophosphate (dTMP). When 5-FU is administered, dTMP becomes scarce, causing rapidly dividing cancer cells to experience thymine-less death [15].

The results of this study confirm our theory that satellite cysts and remnants of keratocyst epithelium, which are assumed to be the root of recurrence, can be efficiently eliminated by topical application of 5-FU to the wound cavity right after curettage and peripheral ostectomy. Topical 5-FU acts differently from MCS or liquid nitrogen, which both produce nonselective tissue necrosis. Three different enzymes, i.e., TS, thymidine phosphorylase (TP), and dihydropyrimidine dehydrogenase (DPD), have an impact on the pharmacologic response of 5-FU [16]. Dihydropyrimidine dehydrogenase is an enzyme involved in uracil and thymidine catabolism and is responsible for the breakdown of 5-FU into its excretory metabolites [17]. Increases in TS messenger ribonucleic acid (mRNA) have been shown to be a marker of resistance to 5-FU [18]. Increased expression of TP in the epithelial lining of inflamed OKCs may encourage the conversion of 5-FU to active metabolites, such as fluorodeoxyuridine monophosphate, and thus enhance the destruction of microscopic satellite cysts unintentionally left behind after surgical removal and the remaining epithelial lining of OKCs. Inflammation-inducing techniques such as intraoperative enucleation and curettage, marsupialization, and previous incisional biopsy may boost the effectiveness of 5-FU treatment of OKCs with low TS and high TP expression in inflamed OKCs. When applied topically to the skin within the first 24 hours, 5-FU has been reported to cause an intense inflammatory response [6]. However, increased DPD expression in the epithelium lining of inflamed OKCs may reduce the effectiveness of 5-FU treatment due to the conversion of 5-FU into inactive metabolites [6].

The availability and technical simplicity of the 5-FU application may make it preferable to other supplementary therapies like MCS or liquid nitrogen, in addition to offering focused antiproliferative therapy for OKC treatment. Simply coated onto ribbon gauze, the 5% 5-FU is placed into the bone cavity in such a way that it may be easily removed 24 hours after surgery. On the other hand, the administration of Carnoy's solution significantly lengthens the duration of the operation due to the measures needed to avoid damaging nearby tissues. The 5-FU-coated gauze should be used carefully to make contact with all surfaces of the wound cavity. The OKC might reoccur in areas that lack exposure to the 5-FU gauze. Also, 5-FU is not known to be neurotoxic, and the size and location of the OKC may be the reason for paresthesia rather than interaction with 5-FU. We follow a protocol that involves applying 5-FU to gauze for 24 hours following peripheral ostectomy and enucleation. To cease tissue exposure to 5-FU, the gauze is removed intact and the bone cavity is irrigated.

The pathogenesis of OKC involves genes such as PTCH1, PTCH2, and SUFU, which are associated with the SHH pathway [19]. The similarities between OKC and BCC at the molecular level suggest the potential use of 5-FU, a drug known to inhibit SHH and induce apoptosis in hepatocellular carcinoma cells. This case report highlights the successful management of a keratocystic lesion using topical application of 5-FU based on its molecular pathogenicity and similarities with BCC. Further research and clinical studies are needed to validate the efficacy of this approach in treating OKC.

## Case presentation

A 38-year-old patient reported to the department with the chief complaint of pain and ulceration in the lower right back region of the jaw for two weeks. The patient was asymptomatic for one to two weeks before she felt pain in the right lower back region of her jaw. The pain was described as sharp, continuous, and radiating to the preauricular region and temporal region. The pain was aggravated by mastication and relieved upon taking medication. The patient also gave a history of ulceration in the same region for three to four days.

The patient's dental history revealed that she underwent surgery in the left lower posterior region of the jaw five years ago at our hospital (a biopsy-proven case of OKC in the left mandible). Also, the patient underwent the extraction of teeth no. 45 to 48 (premolar to the third molar) and no. 35 to 37 (premolar to the second molar) six to seven years ago.

Extraoral examination showed no remarkable facial asymmetry or swelling in the region. Tenderness on palpation in the right-angle region was evident. No signs of paresthesia were present. Intraoral examination showed alveolar ridge tenderness on palpation. No expansion of the buccal and lingual cortical plates was felt on palpation. Ulceration was seen in the 48th region (third molar region), and paresthesia was absent. The patient was partially edentulous in that region.

**Figure 1:**
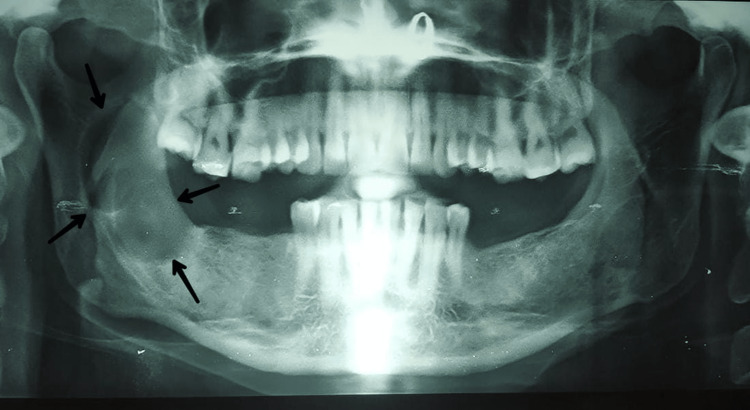
An OPG showing a large well-defined unilocular radiolucency extending from the right ascending ramus region to the subcondylar region anteroposteriorly OPG: Orthopantomagram

**Figure 2:**
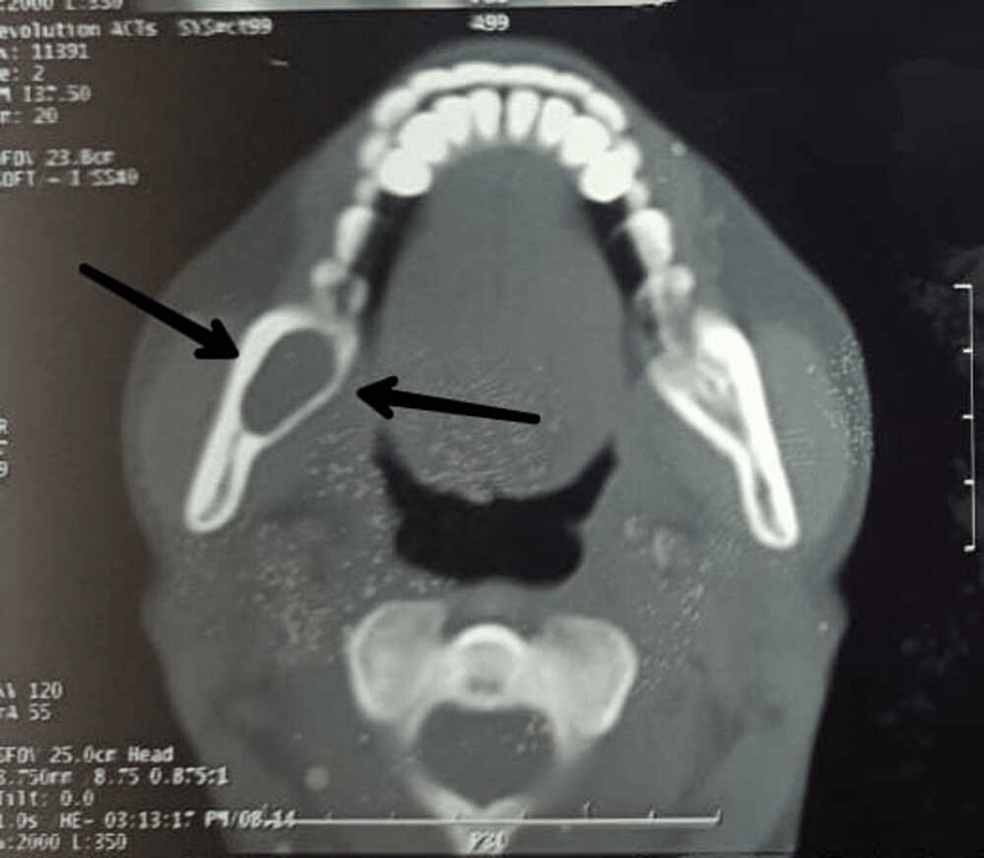
Axial view showing well-defined radiolucency in the right ramus region

**Figure 3:**
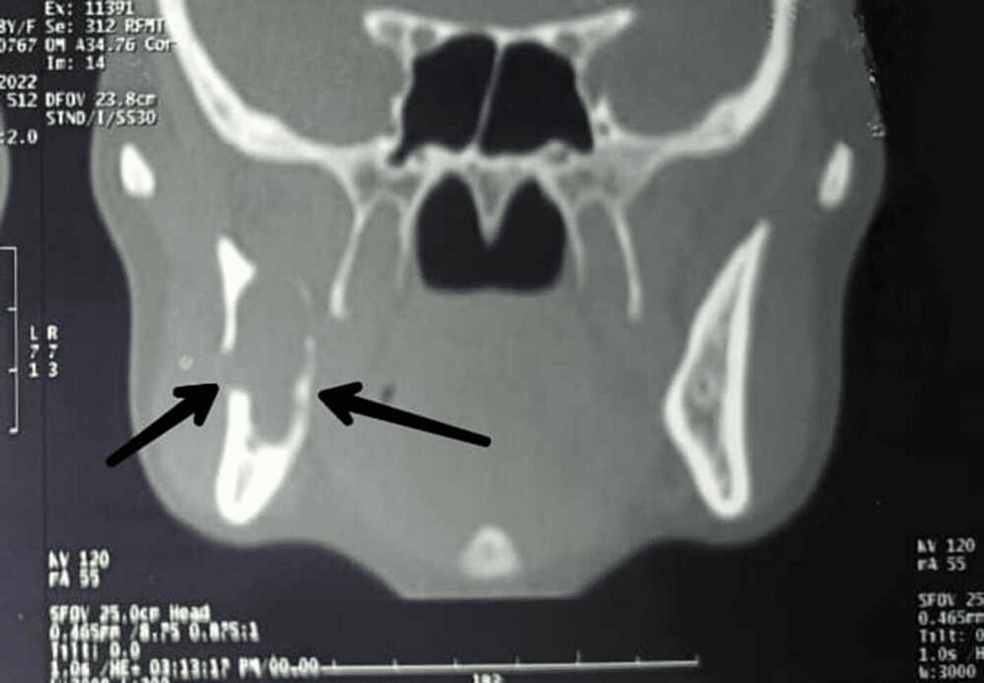
Coronal view showing the thinning and distortion of the cortical plates and its expansion

**Figure 4:**
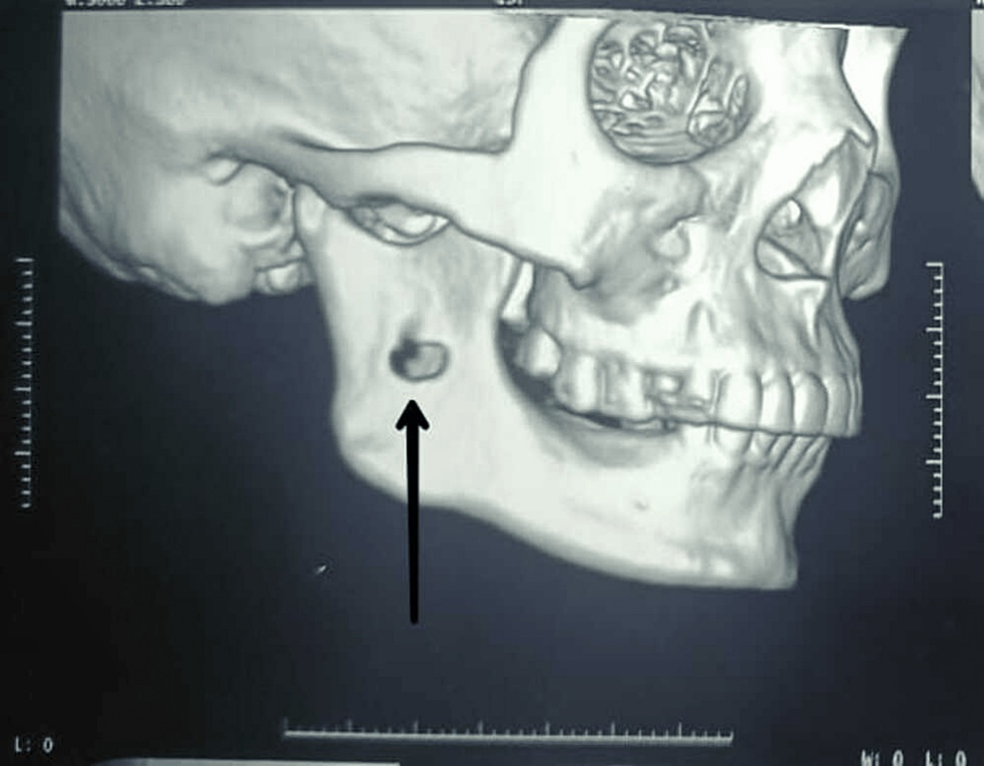
A 3D CT showing perforation at the right ramus region

A preoperative orthopantomagram (OPG) revealed an extension of the lesion from the right ascending ramus region to the subcondylar region (Figure 1). A CT revealed the expansion and thinning of buccal and lingual cortical plates and their perforation in the ramus region (Figures 2-4). 

Enucleation was done under general anesthesia. Exposure was made through an intraoral vestibular incision in the molar region that extended to the ascending ramus region. Access to the cyst was made through a perforation on the ramus, which was gradually extended by using a round bur. Enucleation and debridement of the cystic cavity were done. A sterile ribbon gauze soaked in topical 5-FU was inserted into the cavity after enucleation of the lesion. The wound was closed, leaving only a small opening for the removal of the gauze soaked in 5-FU that was inserted into the cystic cavity after enucleation. The gauze's distal end was left exposed for about 1 cm to facilitate its removal 24 hours after surgery. This was followed by weekly iodoform gauze dressings for six months until the cavity decreased in size with new bone formation. New bone regeneration sites were found in the radiographic follow-up after three months.

The biopsy specimen revealed para-keratinized, stratified, and corrugated squamous epithelium with a thick five- to six-cell layer lining the cystic lumen. The basal cell layer was arranged in a palisading pattern resembling a tombstone. The epithelium was detached from connective tissue. The connective tissue was moderately collagenous, with a few areas of focus showing blood vessels lined by endothelial cells and extravasated RBCs. Histopathological impressions indicated it was an OKC.

**Figure 5:**
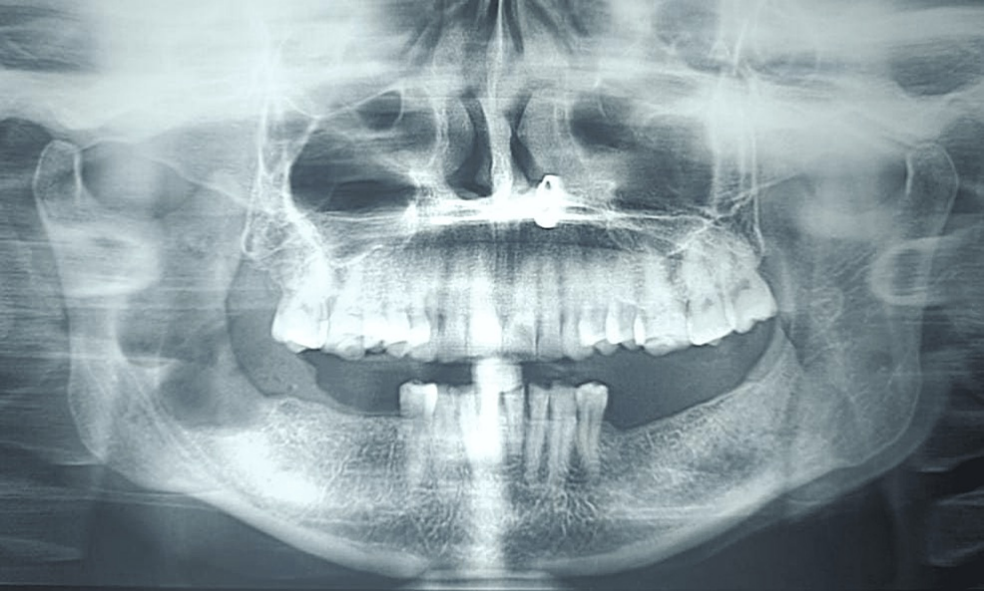
Postoperative OPG on day 1 shows a radiolucent cavity and a single bony septum projecting in the ramus region OPG: Orthopantomagram

**Figure 6:**
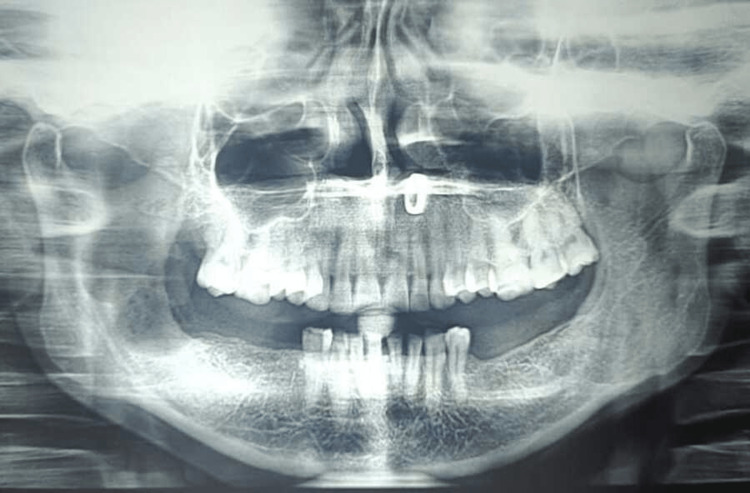
Postoperative OPG in the third month shows a slight radiolucent cavity with evidence of bone regeneration at the periphery OPG: Orthopantomagram

**Figure 7:**
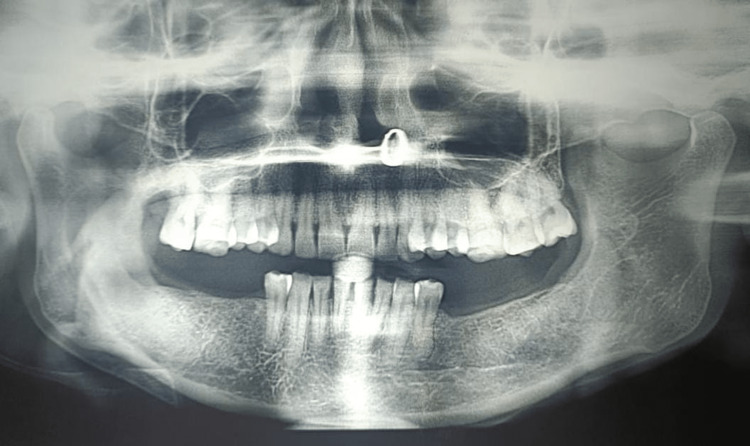
Postoperative OPG in the sixth month reveals a mixed radiopaque-radiolucent region in the ramus-subcondylar region indicative of bone regeneration OPG: Orthopantomagram

**Figure 8:**
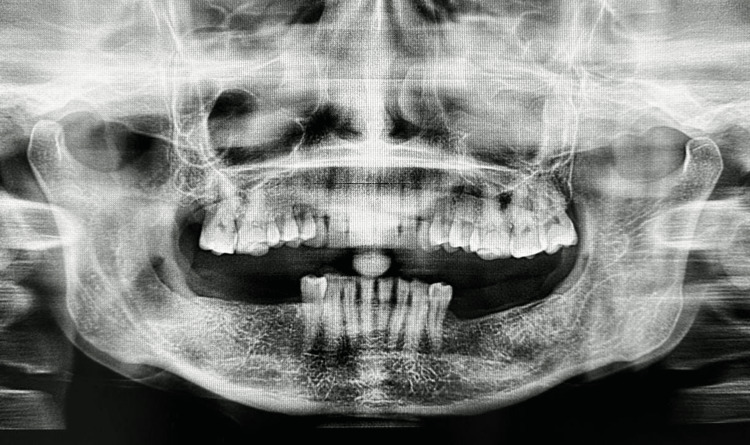
Postoperative OPGin the 12th month depicts evidence of bony trabecular patterns in the region and no signs of recurrence OPG: Orthopantomagram

Without showing any signs of recurrence, the patient was consistently followed up on the third, sixth, and 12th months (Figures 5-8). Radiographic assessment for new bone regeneration and recurrence was done through OPGs.

## Discussion

The keratocystic lesion, among various odontogenic cysts, is unique due to its aggressive nature and high recurrence rate, similar to tumors. The main reasons for OKC recurrence include a higher level of cell proliferative activity in the epithelium, budding in the basal layer of the epithelium, parakeratinization of the surface layer, supraepithelial split, subepithelial split, the presence of epithelial remnants or cell rests, and daughter cysts.

Various treatment modalities have been proposed for OKC. Enucleation with different techniques (primary closure, packing, chemical fixation, cryosurgery), marsupialization alone or followed by enucleation, and resection are among the options mentioned in the literature [14]. The choice of treatment depends on the size, location, and characteristics of the cyst.

The use of marsupialization as a treatment option for OKC has shown high success rates and is considered a conservative approach with low morbidity [2]. This technique helps reduce the aggressive behavior of the cyst by exerting osmotic tension on the surrounding tissues. Marsupialization, performed in two stages, ensures complete removal of the cyst without causing harm to nearby structures and has been associated with lower recurrence rates.

Controversies surrounding OKC treatment arise due to its diverse nature and high recurrence rates. Simple enucleation without additional measures has been associated with recurrence rates ranging from 17% to 56% [2,14]. However, the addition of Carnoy's solution in the cystic cavity after enucleation for a brief period has significantly reduced the recurrence rate to 1.6%, comparable to the rates seen with more invasive resection procedures but without the associated morbidity [2].

Dashow et al. [7], in their study, found that simple enucleation and curettage with Carnoy's solution result in a high recurrence rate of 35%. Chemical curettage with MCS has shown less effectiveness compared to the original solution in reducing recurrence rates. However, a recent retrospective study highlighted the success of using MCS as an adjuvant to enucleation and curettage, and peripheral ostectomy for OKC treatment [2].

The study conducted by Ledderhof et al. provided valuable insights into the management of OKC using Carnoy's solution and 5-FU [20]. They reported that patients treated with MCS experienced an 18% recurrence rate within an average follow-up period of 26.3 +/- 1.8 months. However, there were no recurrences observed in patients treated with 5-FU. This suggests that 5-FU may be a more effective treatment option for OKC compared to Carnoy's solution [14].

It is important to note that the use of Carnoy's solution in OKC treatment was associated with delayed wound healing. Although Carnoy's solution showed some efficacy in reducing the recurrence rate, its potential drawbacks should be considered, especially in terms of postoperative healing and patient comfort. In comparison to MCS therapy, 5-FU is also recommended to effectively reduce postoperative inferior alveolar nerve paresthesia. Furthermore, Balamurgun's report highlighted 5-FU as a trendsetter in the treatment of OKC [21]. This indicates that 5-FU has gained recognition as a promising treatment modality for OKC, potentially offering improved outcomes and reduced recurrence rates.

Overall, these studies and histological classifications contribute to the ongoing understanding of OKCs and provide insights into the selection of treatment modalities that may offer improved outcomes and reduced recurrence rates for patients with this unique and challenging odontogenic cyst.

## Conclusions

It is important to establish protocols for the early diagnosis of OKCs to prevent the need for radical treatments that may result in substantial tissue loss. Long-term follow-up with periodic radiographic examinations is crucial to monitoring the condition and detecting any potential recurrence. With the emergence of newer treatment modalities, such as the application of 5-FU, the recurrence rate of OKC has decreased compared to traditional methods like Carnoy's solution.

From the results of our study and based on various reviews of the literature, 5-FU may be considered ideal due to its availability, technical simplicity, low cost, decreased operating time, reduced morbidity, and inhibition of recurrence. Moreover, the literature also states that the application of 5-FU caused minimal nerve injuries, infection, and swelling, with no recurrence and no compromise in aesthetics or function. Our case too, showed no complications postoperatively. In summary, early identification, utilization of conservative approaches, and long-term monitoring are crucial in the management of OKC. Though the application of 5-FU shows promising results in reducing recurrence rates, continued research and follow-up studies are necessary to further understand and optimize the management of OKC.
